# Oral inflammatory load predicts vascular function in a young adult population: a pilot study

**DOI:** 10.3389/froh.2023.1233881

**Published:** 2023-08-18

**Authors:** Ker-Yung Hong, Avin Ghafari, Yixue Mei, Jennifer S. Williams, Dina Attia, Jourdyn Forsyth, Kevin Wang, Trevor Wyeld, Chunxiang Sun, Michael Glogauer, Trevor J. King

**Affiliations:** ^1^ Department of Kinesiology, Faculty of Science, McMaster University, Hamilton, ON, Canada; ^2^ Faculty of Dentistry, University of Toronto, and Dental Oncology, Princess Margaret Cancer Centre, Toronto, ON, Canada; ^3^ Department of Health and Physical Education, Faculty of Health, Community and Education, Mount Royal University, Calgary, AB, Canada

**Keywords:** vascular function, cell count, neutrophils, periodontal disease, oral inflammatory load, rinse test, flow-mediated dilation

## Abstract

**Background:**

The periodontium is a highly vascularized area of the mouth, and periodontitis initiates negative functional and structural changes in the vasculature. However, mild oral inflammation, including levels experienced by many apparently healthy individuals, has an unclear impact on cardiovascular function. The purpose of this pilot study is to investigate the effects of objectively measured whole mouth oral inflammatory load (OIL) on vascular function in apparently healthy individuals.

**Methods:**

In this cross-sectional and correlational analysis, we recruited 28 young (18–30 years) and systemically healthy participants (16 male, 12 female). Using oral neutrophil counts, a validated measure for OIL, we collected participant's mouth rinse samples and quantified OIL. Blood pressure, arterial stiffness (pulse-wave velocity) and endothelial function (brachial artery flow-mediated dilation) were also measured.

**Results:**

Only oral neutrophil count significantly predicted flow-mediated dilation % (*p* = 0.04; *R*^2 ^= 0.16, β = − 1.05) and those with OIL levels associated with >2.5 × 10^5^ neutrophil counts (*n* = 8) had a lower flow-mediated dilation % (6.0 ± 2.3%) than those with counts associated with gingival health with less than 2.5 × 10^5^ neutrophil counts (10.0 ± 5.2%, *p* = 0.05). There were no significant predictors for arterial stiffness.

**Conclusion:**

We found that OIL was a predictor of reduced flow-mediated dilation. An impairment in flow-mediated dilation is an indicator of future possible risk of cardiovascular disease—one of the leading causes of death in North America. Therefore, this study provides evidence for the importance of oral health and that OIL may impact endothelial function.

## Introduction

Inflammation of the gingival tissues occurs even before the onset of periodontal disease. If left untreated, gingival inflammation can progress to periodontal disease. Periodontal disease affects up to 90% of the world's adult population (1), and the presence of periodontitis has been shown to be associated with the development of cardiovascular diseases (CVD) such as myocardial infarction and stroke (2–5). A proposed mechanism for this association is the infiltration of inflammatory mediators and bacteria into the systemic circulation via the vasculature in the periodontium. As a result, increases in proinflammatory cytokines (2, 6), periodontal bacteria (7), and uncontrolled production of nitric oxide (NO) (2, 8) are seen in the vascular system which can lead to detrimental structural and functional changes throughout the body. Therefore, good oral hygiene is often included as a crucial component of a healthy lifestyle. We have developed a non-invasive oral rinse test that allows for the measurement of oral inflammatory load (OIL) through the quantification of neutrophils with in a 30 s oral rinse (9, 10). This enables for the measurement of oral inflammation in a non-dental setting and allows for clinical studies in non-dental settings regarding relationships between oral health and systemic health (11, 12).

Most research linking oral health to CVD has investigated severe forms of oral inflammation that accompany periodontitis (6) in older, physically inactive populations, and little is known regarding whether less severe oral inflammation (which occurs commonly in young individuals) can impact cardiovascular health. Past studies have used subjective measures to determine the severity of oral inflammation such as questionnaires (13, 14) and clinician diagnosis (3, 15, 16), both of which can cause inter-subject and inter-rater variability and require significant time and costs. However, our use of oral neutrophil counts allows for a rapid and objective grading of oral inflammation (9, 17–19). Furthermore, the previously researched cohorts of middle and older aged adults (3, 6, 7, 15, 16, 20, 21) are more likely to have comorbidities and confounding variables, making it difficult to isolate the relationship between early periodontal disease and vascular function. Examining gingival health in a young, healthy population can attenuate confounding factors.

Early risks for CVD have been observed in young individuals with disease states that elevate inflammation (e.g., obesity, diabetes) (22, 23), thus if mild OIL poses an impairment to cardiovascular health, this might present itself among young and healthy individuals. CVD risk can be detected via traditional indicators (blood pressure, lipids, blood glucose), however an earlier and more direct indicator of artery health can be identified using pulse-wave velocity (PWV) (24) and flow-mediated dilation (FMD) in all ages (25).

Therefore, the purpose of this pilot study was to investigate whether objectively measured levels of OIL can predict early vascular dysfunction in young, healthy males and females. We used a well characterized method of measuring OIL (oral neutrophil count) from a saliva sample, which is rapid, non-invasive, scalar, and objective for determining levels of gingival infammation (9, 17). Vascular health and function were measured using the non-invasive direct assessment of vascular endothelial function via a technique called FMD and arterial stiffness (a measure of artery structure and health) was measured non-invasively via PWV. We hypothesized that greater OIL would predict lower vascular function, indicating increased risk for the future development of CVD.

## Material and methods

### Research design

This cross-sectional and correlational study collected primary data from the assessment of OIL and vascular function in one visit in a young and apparently healthy population with no self-reported history of periodontal disease. Using an objective measure of OIL we predicted vascular function via linear regression. This study was approved by the Hamilton Integrated Research Ethics Board (REB project # 14145) and was conducted in accordance with the Declaration of Helsinki, as revised in 2013. Raw data are available upon request.

### Overview of methodology

After receiving informed consent, participants arrived at the lab having fasted for at least 6 h prior and avoided exercise or consumption of food/drink with alcohol or caffeine for at least 8 h prior to the testing session. Lab times for females were scheduled during their early follicular phase of their menstrual cycle (estimated as the first 2 days following the onset of menstruation or the placebo phase of birth control). Oral contraceptives were the only birth control method included in this study. After height and weight were measured, participants rinsed their mouth with tap water for 10 s to remove any particulate. Subsequently, a 30 s saline mouth rinse was performed, and the resulting mouth rinse samples were collected and stored for later analysis. The participants then laid supine for a minimum of 10 min while instrumented with electrocardiogram (ECG) to measure heart rate. After the 10 min, vascular measures (blood pressure, PWV and FMD) were performed while in the supine position.

### Participant characteristics

Male and female participants between the ages of 18–30 were recruited from the McMaster and Hamilton communities. Participants were recruited via word of mouth, posters, and social media posts. To avoid selection bias, recruitment and participant interaction was not performed by dental professionals, and posters were not placed in a dental setting. Based on previous studies analyzing the effects of gingival inflammation in older adults on vascular function which showed a 4.5% difference (15), a sample size calculation suggested only 10 participants would be required. However, due to expected lower levels of inflammation in our young healthy participant population, we used a sample size of 28 (16 males, 12 females) which was powered to detect a 2.3% difference at a power of 0.8. Any difference greater than 1% is considered to be clinically significant, (26). The participants were healthy, non-smoking individuals with no history of CVD or other comorbidities. Participants did not regularly use any medication that is known to influence the cardiovascular system. Individuals with a history of hypertension or a body mass index (BMI) greater than 30 kg/m^2^ were excluded due to their known independent influences on cardiovascular function.

### Height and weight

Height and weight were used for the calculation of BMI to help us determine risk stratification and study eligibility. A stadiometer (AnthroFlex) was used to measure height and a digital scale (Malama) was used to measure weight.

### Saline mouth rinse

Participants did not consume food or any type of drink (except water) 6 h prior to the oral rinse samples. The participant pre-rinsed their mouth with tap water for 10 s, waited 2 min, and then rinsed their mouth with 10 ml of sterile saline (0.9% sodium chloride) solution (Baxter) for 30 s and expelled the entire rinse into a 20 ml collector tube. The collected samples were fixed to a final 4% formaldehyde to preserve the samples for shipping to the lab for counting. The solutions were kept in a 4 °C refrigerator for a maximum of 2 days before they were analyzed. To count the neutrophils, the rinses were centrifuged. After discarding the supernatant, cell pellets were resuspended by pipette in 500 µl of Hank's Balanced Salt Solution. After suspension, 250 µl of cells were stained with 4 µg of Acridine orange. The samples were then incubated in a dark room at room temperature for 15 min. To establish uniform suspensions following incubation, the samples were resuspended by pipette after incubation. To facilitate accurate counting, the sample were diluted by a factor of 10 prior to counting. Neutrophils were counted visually on a hemocytometer at 200 and 400× final magnification (17).

### Blood pressure

Brachial artery blood pressure was assessed in triplicate using an automated sphygmomanometer (GE Dynamap V100). Participants laid supine while the blood pressure cuff was fitted on their upper left arm. The device is designed to automatically inflate and deflate at one-minute intervals. Mean arterial pressure (MAP) was collected as the product of systolic blood pressure and diastolic blood pressure using the equation:

MAP=[SBP+(2×DBP)]/3.

### Arterial stiffness

Carotid- femoral PWV measured arterial stiffness, which is the gold standard (27). This technique measured the velocity of the arterial pulse along the thoracic and abdominal aorta. As vascular compliance reduces, the vessel walls stiffen which yields a faster PWV. For this non-invasive assessment, participants were asked to lie down in a supine position and were instrumented with a lead 1 electrocardiogram (ECG) and ground. Testing was done in a dimly lit, temperature controlled, and quiet room. After resting for 10 min, a pressure-sensing tonometer (Millar Instruments) was placed against two arterial sites: (1) carotid pulse (neck), and (2) femoral pulse (inner thigh)*.* The tonometers simultaneously detected pulse waves, allowing for the collection of transit time and the distance between the two locations determined velocity. A correction factor of 0.8 was applied to the carotid femoral distance to account for the bisection of the pulse pressure wave up the carotid artery and down the aorta (28). PWV data were collected on Labchart (ADInstruments) and band-pass filtered to determine the foot of the pressure wave. The time between the arrival of the pressure wave at the carotid and femoral arteries was used to calculate PWV for one cardiac cycle. To calculate PWV, the following equation was used:

$$\frac{\left[0.8(\mathrm{Carotid}\;\mathrm{to}\;\mathrm{femoral}\;\mathrm{distance}\;:)\right]}{\mathrm{Pulse}\;\mathrm{wave}\;\mathrm{transit}\;\mathrm{time}}$$

PWV for 20 cardiac cycles were averaged to determine PWV for each participant.

### Endothelial function

In the supine position, a 1 lead ECG was collected. The brachial artery was imaged on the upper right arm using duplex ultrasound, and an occlusion cuff was placed (uninflated) on the lower right arm (29). High resolution ultrasound video recordings were taken using the B-mode ultrasound with the ultrasound probe operating at 7.5–12 Mhz and an insonation angle calibrated to less than or equal to 60 degrees. Each test began with a baseline phase where the brachial artery was imaged for at least 30 s to capture resting artery blood velocity and diameter. Next was the occlusion phase to create an ischemic stimulus. Occlusion involved the rapid inflation of the cuff that was wrapped around the forearm to 200 mmHg using a rapid cuff inflator (E20 Rapid Cuff Inflator and AG101 Air Source; Hokanson, Bellevue, WA). After 5 min, the cuff was released, to produce a rapid increase in blood flow (reactive hyperemia). The arteries were imaged for 3 more minutes to capture peak arterial dilation and the total blood flow stimulus in the reactive hyperemia phase. The ultrasound captured 3 phases: the baseline phase, the 4 min phase (1 min before cuff release), and the cuff release phase.

To analyze FMD, a Sante DICOM Editing program was used to extract the end-diastolic images. The artery diameters were analysed using an automated edge detecting software (Arterial Measurement System, Gothenburg, Sweden). Manual selection was used if the automated edge detection was incorrect. To quantify macrovascular endothelial function, FMD was expressed as a percentage change in artery diameter relative to baseline artery diameter. The following was the formula used to calculate FMD%:

$$\mathrm{FMD}\;(\text{\%})=\frac{\left(\mathrm{Artery}\;\mathrm{Diamete}r_{\mathrm{Peak}}-\mathrm{Artery}\;\mathrm{Diamete}r_{\mathrm{Baseline}}\right)}{\mathrm{Artery}\;\mathrm{Diamete}r_{\mathrm{Baseline}}}\times 100\;\text{\%}$$

The baseline diameter was determined using the average of all diameters from each frame taken at baseline FMD. Note that for some participants, an average of each frame taken 1 min before cuff release were use for the baseline diameter due to unanalyzable ultrasound baseline images or unstable baseline diameters. The peak diameter was found by taking the max from a 3 s average of all frames after cuff release.

Blood velocity was captured via Doppler ultrasound and recorded on Labchart (ADInstruments). Shear rate, the frictional force caused by blood flow, is the stimulus for FMD. The shear rate stimulus was estimated as shear rate area under the curve for the first 30 s following cuff release, and was calculated using the following equations:

$$1.\;\mathrm{Mean}\;\mathrm{Baseline}\;\mathrm{Shear}\;\mathrm{Rate}=\frac{4(\mathrm{Mean}\;\mathrm{Baseline}\;\mathrm{BV})}{\mathrm{Baseline}\;\mathrm{Artery}\;\mathrm{Diameter}}$$

$$2.\;\mathrm{RH}\;\mathrm{Shear}\;\mathrm{Rate}=\frac{4(\mathrm{mean}\;\mathrm{RH}\;\mathrm{blood}\;\mathrm{velocity})}{\mathrm{Artery}\;\mathrm{Diameter}}$$

3.ShearRateAreaUndertheCurve=(30smeanRHShearRate−BaselineShearRate)30sec

The baseline blood velocity used for Equation 1 was a 30 s average of blood velocity at baseline and the RH blood velocity used for Equation 2 was a 30 s average of blood velocity right after cuff release.

### Statistical analyses

Normality was confirmed via Shapiro–Wilk tests (FMD, PWV). A correlation was run between neutrophil count and FMD%, PWV, Shear rate, MAP, and BMI to determine variables to include in a multi linear regression. Only FMD% correlated significantly with oral neutrophil count, therefore a multi-linear regression was not performed in the primary analysis, and a simple linear regression was used to predict FMD% from oral neutrophil count.

In an exploratory analysis, participants were split into “high-OIL” and “low-OIL” based on a cutoff of 2.5 × 10^5^ neutrophil counts. This cutoff was determined by visual inspection of the figures where there were two clear clusters of neutrophil counts above and below 2.5 × 10^5^ neutrophil counts. A *T*-test was performed between these groups. We were underpowered to split participants into tertiles. Additionally, a *T*-test was used to compare males and females to identify sex differences in FMD% and neutrophil count. Alpha was set at *p* < 0.05.

## Results

The participant characteristics are summarized in Table 1. No significant differences were found in the reported characteristics between the males and females.

**Table 1:** Baseline characteristics.

Anthropometric Variable	Male (*n* = 16)	Female (*n* = 12)	All (*n* = 28)
Age, *y*	21 ± 1	22 ± 3	22 ± 2
Resting HR, *bpm*	61.5 ± 7.7	62.8 ± 10.5	62.1 ± 8.9
Systolic BP, *mmHg*	119.6 ± 10.9	111.3 ± 9.1	116.0 ± 10.8
Diastolic BP, *mmHg*	62.7 ± 4.9	65.6 ± 7.5	64.0 ± *6.2*
MAP, *mmHg*	82.3 ± *6.0*	81.5 ± 8.7	82.0 ± 7.2
BMI, *kg/m^2^*	24.4 ± 2.8	23.4 ± 4.0	24.0 ± 3.3
FMD, *%*	8.5 ± 5.4	9.3 ± 4.3	8.9 ± 4.9
PWV, *m/s*	6.7 ± 1.8	7.0 ± 2.3	6.8 ± 2.0

HR, Heart Rate; BP, Blood Pressure; MAP, Mean Arterial Pressure; BMI, Body Mass Index; FMD, Flow-Mediated Dilation; PWV, Pulse Wave Velocity. Data are mean ± standard deviation.

### Oral neutrophils

Oral neutrophil count ranged from 1.2 × 10^4^ to 6.5 × 10^5^ neutrophil counts with a mean of 2.09 × 10^5^ (±1.9 × 10^5^) counts. The majority of neutrophil count values indicated that most participants had mild OIL.

### Arterial stiffness

**Figure 1:**
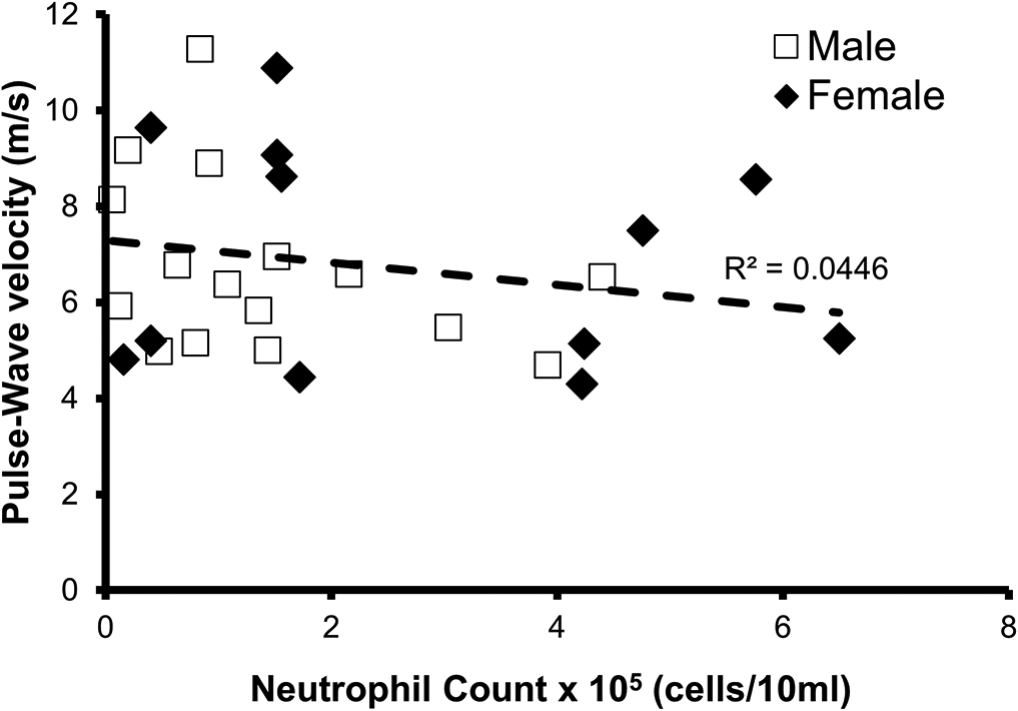
Relationship between oral neutrophil count and pulse-wave velocity (PWV). There was not a significant relationship between neutrophil count and PWV in all participants (*n* = 28, *p* = 0.3, *R*^2^ = 0.045).

The relationship between PWV and oral neutrophil count is shown in Figure 1. There was not a significant relationship between oral neutrophil count and PWV (*p* = 0.3; *R*^2^ = 0.045). When split into “high OIL” and “low OIL” at 2.5 × 10^5^ neutrophil counts, there was no significant relationship either (*p* = 0.1).

### Endothelial function

**Figure 2:**
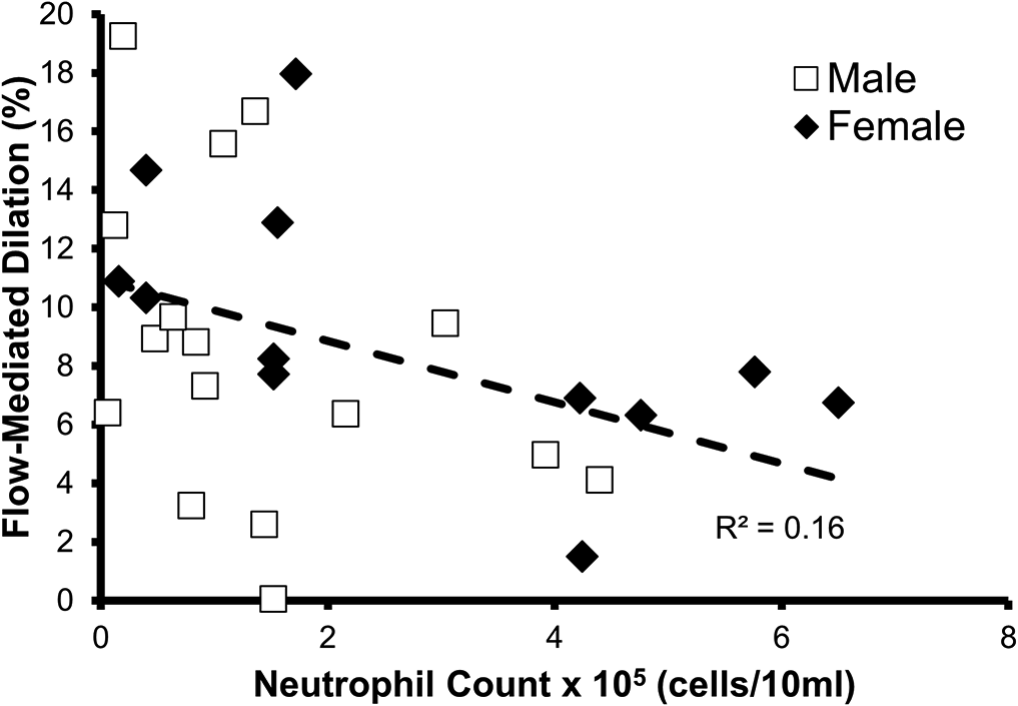
Relationship between oral neutrophil count and flow-mediated dilation % (FMD%). A statistically significant relationship was found to exist between neutrophil count and FMD% (*n* = 28, *p* = 0.04, *R*^2^ = 0.16).

**Figure 3:**
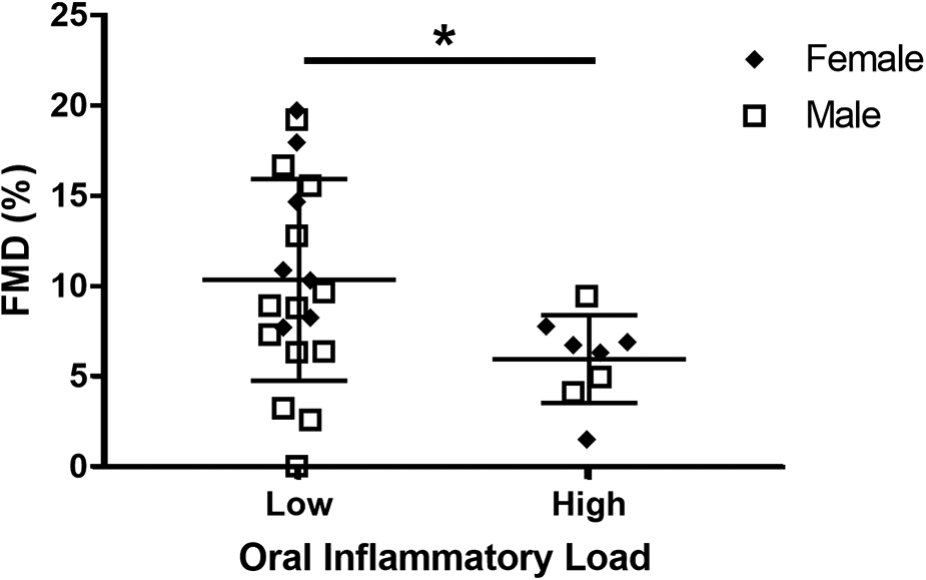
Comparison between high oral inflammatory load (OIL) and FMD% vs. low OIL and FMD%. FMD% was significantly higher in high OIL participants (*n* = 8) than in low OIL participants (*n* = 20, *p* = 0.05).

The relationship between FMD% and oral neutrophil count is shown in Figure 2. FMD% was shown to be inversely and significantly associated (*p* = 0.04; *R*^2 ^= 0.16) with oral neutrophil count. When split into “high” and “low” OIL at 2.5 × 10^5^ neutrophil counts, those with high-OIL (*n* = 8) had significantly lower FMD (6.0 ± 2.3%) than those with low-OIL (*n* = 20, 10.0 ± 5.2%, *p* = 0.05) (Figure 3).

**Figure 4:**
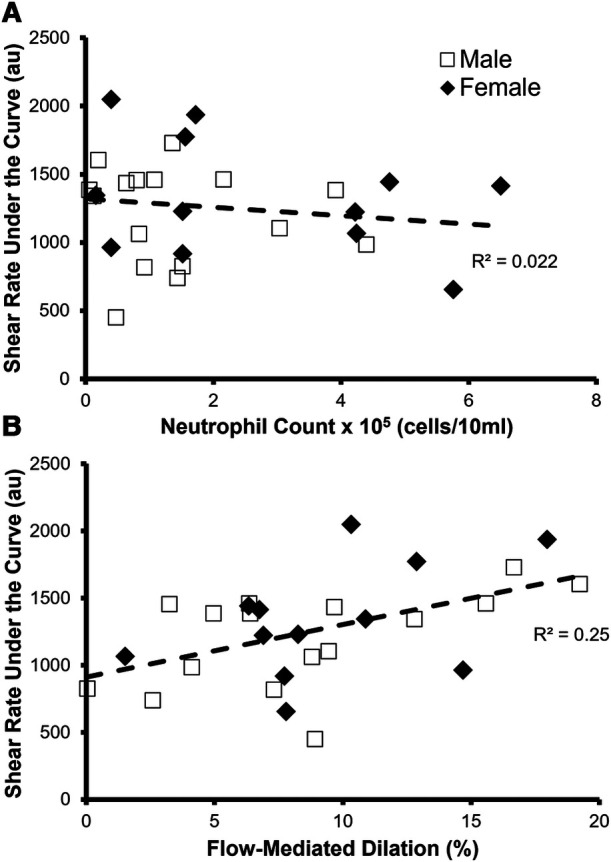
Identifying factors that predict the stimulus for FMD—shear rate area under the curve. (**A**) There was not a significant relationship between neutrophil count and shear rate under the curve in all participants (*n* = 28, *p* = 0.5, *R*_2_ = 0.022). (B) Relationship between flow-mediated dilation % (FMD%) and shear rate under the curve in all participants (*n* = 28). FMD% significantly predicted shear rate under the curve (*p* = 0.007, *R*_2_ = 0.25).

As expected, shear rate area under the curve correlated with FMD% (*p* = 0.007 *R*^2 ^= 0.25) (Figure 4A). Furthermore, neutrophil count did not correlate with the shear rate area under the curve (*p* = 0.5, *R*^2 ^= 0.022) (Figure 4B), which is the stimulus for FMD, suggesting that any relationship between neutrophil count and FMD% was due to changes in endothelial function.

In an exploratory analysis, a multi-linear regression was run using MAP, BMI, and oral neutrophil count to predict for FMD%. We found that only oral neutrophil count trended towards significance when predicting for FMD% (*p* = 0.06).

### Sex differences

We were not adequately powered to observe sex differences between female participant's oral neutrophil count and FMD% (*p* = 0.04, *R*^2 ^= 0.36) and between male's neutrophil count and FMD% (*p* = 0.2, *R*^2 ^= 0.14).

## Discussion

**Figure 5:**
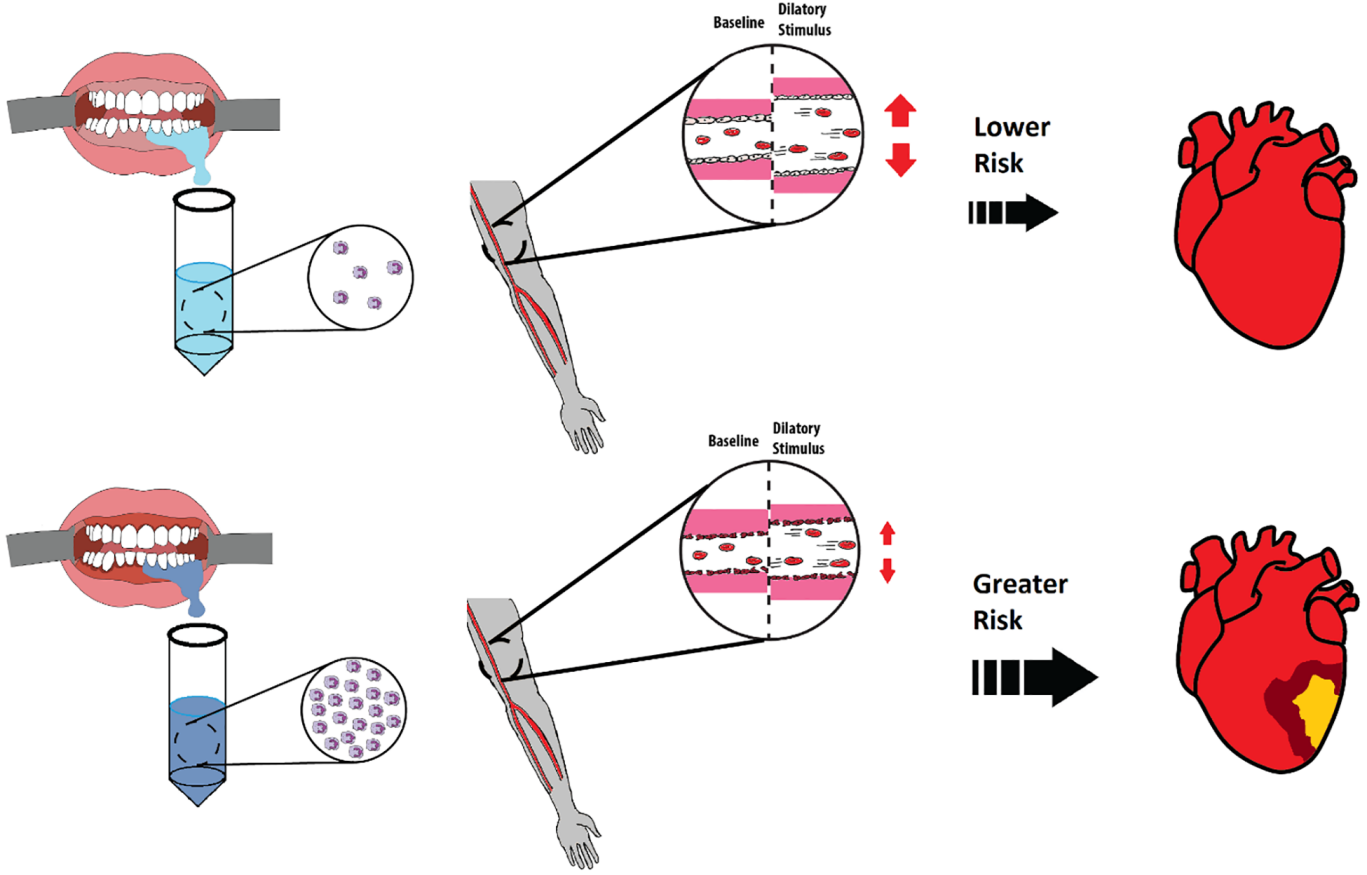
Comparison of healthy and inflamed gingiva and implications each has on future development of cardiovascular diseases (CVDs). Healthy gingiva has fewer neutrophils in saliva (left top) whereas inflamed gingiva has an abundant number of neutrophils in saliva (left bottom). A healthy and functional endothelium (middle top) will have a greater flow mediated dilation % in response to a dilatory stimulus as opposed to an unhealthy endothelium (middle bottom). This study identified a relationship between OIL and reduced flow mediated dilation in young and healthy participants, indicating a possible elevated future risk of developing CVD compared to healthy gingiva.

This study sought to determine if OIL levels relate to vascular endothelial function and artery structure in apparently healthy young participants. We found that higher oral neutrophil count predicted attenuated FMD of the brachial artery, indicating poorer endothelial function. Endothelial dysfunction is an early risk factor for CVD and therefore these findings suggest that even mild OIL may increase future risk of developing CVD (Figure 5). Furthermore, our findings support a potential mechanistic link between OIL and CVD via endothelial dysfunction. However, contrary to our hypothesis, OIL did not predict arterial stiffness.

### Mechanistic relationship between oral bacteria and arteries

The junction epithelium separates the sulcus and the delicate blood vessels in the underlying periodontium (30). The junction epithelium is permeable, and allows for diffusion of metabolic products (e.g., endotoxins, chemotactic agents, inflammatory cytokines, and antigens) from the plaque/biofilm located in the sulcus (31, 32). Oral inflammation is associated with the level of plaque bacteria present in the gingiva (33) and we measured this OIL in this study using oral neutrophil count. The metabolic products in the plaque bacteria can move via diffusion into the blood stream thereby enhancing systemic inflammation (34). These extravascular metabolic products can enhance systemic inflammatory cytokines (34) and may lead to disruptive effects on the blood vessel's endothelium, including the reduced ability for the endothelium to produce nitric oxide (6, 7, 30, 31). These effects can cause endothelial dysfunction impairing the ability of the blood vessels to dilate in response to shear stress. Prolonged endothelial dysfunction is detrimental because the nitric oxide that is released in a healthy endothelium is a mechanism that protects against the development of atherosclerosis. Furthermore, a functional endothelium allows for blood flow maintenance in diseased arteries.

In a healthy functioning artery, the vascular endothelium senses an increase in blood flow through the artery via shear stress. This shear stress stimulates the release of vasoactive molecules, including nitric oxide, which diffuse into the smooth muscle causing vasodilation. However, a dysfunctional endothelium produces less vasodilators in response to the same amount of shear stress, resulting in less vasodilation (35–37). We measured shear rate, which is a surrogate measure of shear stress, to confirm that OIL was impairing the vasodilatory response, and not the shear stress stimulus, because less shear rate would result in a lower FMD. However, our data indicate that oral neutrophil count did not predict shear rate, and therefore a reduced shear rate was not the cause of impaired FMD. Although we did not directly test mechanisms, the attenuated vasodilation may be caused by the elevated level of systemic inflammation because of the increased diffusion of extravascular metabolic products from the oral plaque (6, 8, 15, 32, 34).

We did not observe any relationship when comparing oral neutrophil count to PWV, an index of arterial stiffness, indicating that longer term impacts of OIL on artery structure had not occurred. This observation is in line with previous studies (16, 21) that include older individuals, and suggests that OIL has little impact on artery structure in young individuals as well.

### Strengths and limitations

Many studies have provided associations between progressed periodontal disease and vascular function (2, 6–8, 20, 21, 32), however the effect of mild gingival inflammation has been inconclusive (3, 13, 33, 34, 38). Most previous studies used subjective questionnaires (13, 14) as well as assessments from different clinicians (3, 15, 16), making the levels of gingival inflammation not standardized and/or difficult to stratify among different levels of mild inflammation. In this study, we utilized an objective measure of oral neutrophil counts which are analogous to measuring neutrophil counts in blood to determine the level of OIL (9, 17–19). It is interesting to note that even within levels of oral inflammation that are below the levels typically seen in gingivitis (9), there was still a significant impact on endothelial function. Higher, and therefore more clinically relevant levels of oral inflammation may have an even greater association with vascular dysfunction. Another strength of this study was the use of healthy young adults as the population. The population were young, non-smoking, healthy adults, with no history of CVD. They were also within the healthy range of BMI and had no other comorbidities unlike previous studies (3, 13–16, 21). Our preclusion of participants with conditions such as history of periodontal disease relied on self-reported information, and we can not exclude the possibility of self-report and information bias. However, the present study's young healthy population includes many participants with objectively measured low-OIL yielding a better representation of a healthy gingiva and controlling for many confounding variables that may affect the association of oral inflammation and vascular function.

Due to the nature of the cross-sectional design, future research should perform oral care interventions with a washout design to alleviate untested between-participant differences. Though the sample size of this study was smaller than previous studies (3, 13–15), this is likely not a concern as we were powered to see expected changes, and we did see significant differences. Although this study looked at levels of gingival inflammation, future studies can incorporate more participants with clinical gingivitis (previous study mean 8.8 × 10^5^ neutrophils (9, 17), confirmed via manual pocket-depth testing and/or other salivary biomarkers for gingivitis. This study did not measure established clinical assessments of periodontal disease such as pocket-depth testing, radiographic examinations, or bleeding index, which limits its clinical validity. However, this study provides evidence that OIL at low levels that would not previously be considered clinically significant still may have an impact on cardiovascular health.

## Conclusion and significance

We found that mild OIL, measured by oral neutrophil count, was inversely related to FMD% in apparently healthy individuals. An impairment in FMD is an indicator of future possible risk of CVD (39). Therefore, this study provides evidence that oral health may have impact on cardiovascular health, even among young healthy individuals.

## Data Availability

The raw data supporting the conclusions of this article will be made available by the authors, without undue reservation.
